# Supplementary material for: Empirical methods for controlling false positives and estimating confidence in ChIP-Seq peaks
Source: BMC Bioinformatics. 2008 Dec 5;9:523. doi: 10.1186/1471-2105-9-523 (PMC2628906; doi:10.1186/1471-2105-9-523)
Supplement: Additional File 1 — A variety of html documents from the USeq web site detailing the available applications, their best usage, output file type descriptions, command line menus, etc. [file 1471-2105-9-523-S1.zip › USeqUserGuides/JavaDoc.zip!/JavaDoc/index-files/index-3.html]

 
 
 
 
 
 
C-Index
 


 

 
function windowTitle()
{
    parent.document.title="C-Index";
}
 
 
 

 

 


 
   
  
 
 
 
   
 
   
          Overview     
        Package   
        Class   
        Use   
          Tree     
          Deprecated     
       Index    
          Help     
   
 
 
  
 
 
 

 
  
   PREV LETTER   
   NEXT LETTER    
  
    FRAMES     
   NO FRAMES     
  
    All Classes  ');
  }
  //-->
 
 
    All Classes  
 


  
 
 
  
 

 A   B   C   D   E   F   G   H   I   J   L   M   N   O   P   Q   R   S   T   U   V   W   X   Z   
    
 C  
 
   CalcBkgrndRatios   - Class in  trans.bkgrnd  Calculates a correlation coefficient between ratio scores and tm or gc 
 for a set of CelMapper converted cel/bpmap files.   CalcBkgrndRatios(String[])   - 
Constructor for class trans.bkgrnd. CalcBkgrndRatios 
  
   calcDefaultNearestNeighborTm(String)   - 
Static method in class util.bio.calc. NucleicAcid 
 Calculates a nearest neighbor Oligo Tm at 50nM oligo, 50mM Salt, also corrects case
 for real method.
   calcFDR(double)   - 
Method in class trans.roc. RocWindowScanner 
 Scans an array of Window intersecting with positives to calculate an FDR at a given threshold.
   CalcGCAndTransform   - Class in  trans.misc  Parses out treatment and control oligo info from intervals, calculate GC and transform ratios based on GC corrector, 
 see   TransformRatios  .   CalcGCAndTransform()   - 
Constructor for class trans.misc. CalcGCAndTransform 
  
   CalcGCRatiosFromIntervals   - Class in  trans.misc  Parse out treatment and control oligo info from intervals, calculate GC and ratios to look for bias.   CalcGCRatiosFromIntervals()   - 
Constructor for class trans.misc. CalcGCRatiosFromIntervals 
  
   calcMaxMedianAbsoluteDifference(float[][])   - 
Static method in class util.gen. Num 
 Calculates the maximum median absolute difference between arrays of float where int[oligo index number][oligo intensity measurements]
   calcMeanMedianAbsoluteDifference(float[][])   - 
Static method in class util.gen. Num 
 Calculates the mean of the median absolute differences between arrays of float where float[oligo index number][oligo intensity measurements]
   calcSmallSample10Log10PValue(float, int)   - 
Method in class trans.main. WilcoxonSignedRankTest 
 For calculating a -10Log10(pValue) for small sample sizes (N 5-9).
   calculateAdditivePairCorrelation()   - 
Method in class util.gen. PearsonCorrelation 
 Following loading of this class using the addPairsToCorrelate(),
 call this method to calculate to correlation coefficient (R) on the total.
   calculateAndPrintStats(Region[])   - 
Method in class trans.anno. IntersectKeyWithRegions 
  
   CalculateBackgroundStats   - Class in  trans.bkgrnd  For each CelMapper converted cel/bpmap files, this calculates a correlation coeff between pm:mm, pm:tm, pm:gc.   CalculateBackgroundStats(String[])   - 
Constructor for class trans.bkgrnd. CalculateBackgroundStats 
  
   calculateBasicOligoTm(String)   - 
Static method in class util.bio.calc. NucleicAcid 
 Calculate basic Oligo Tm
   calculateBinFDRs()   - 
Method in class trans.roc. MethodTester 
 Runs through all the bins calculating and setting fdrs for each.
   calculateChromControlSpecificMedian(String[])   - 
Method in class trans.cel. QuantileNormalization 
 Given chromosome names (e.g.
   calculateControlStats(int[][][], double, double, double, double)   - 
Method in class trans.qc. CelFileStats 
 Calculates the median value for each of the different classes of controls.
   calculateEffectiveMandN(double, double, double, double)   - 
Static method in class gata.main. GATAUtil 
 Calculates effective m and n using BLAST parameters, returning int[m', n']
   calculateEffectiveMandN(double, double, double, double)   - 
Static method in class util.bio.calc. Alignment 
 Calculates effective m and n using BLAST parameters, returning int[m', n']
   calculateEValue(double, double, int, double, double)   - 
Static method in class gata.main. GATAUtil 
  
   calculateEValue(double)   - 
Method in class gata.plotter. ToolsFrame 
  
   calculateEValue(double, double, int, double, double)   - 
Static method in class util.bio.calc. Alignment 
 Calculates an E value given a variety of alignment parameters
   calculateFractionGC(String)   - 
Static method in class util.bio.calc. NucleicAcid 
 Calculates a GC concentration, case insensitive.
   calculateFractionGCContent(int, int)   - 
Method in class trans.roc. ScoreParsedBars 
 Calculates the gc content.
   calculateFudgeFactor(int)   - 
Method in class trans.main. SamChromosomeData 
  
   calculateGCContent()   - 
Method in class trans.anno. Region 
 Returns the fraction of GC provided the Region.gcContent[] is loaded.
   CalculateGeneExtensions   - Class in  trans.misc  Finds isolated genes that are expressed and using Huber's segmentation algorithm, slices it into three and prints the extension.   CalculateGeneExtensions(String[])   - 
Constructor for class trans.misc. CalculateGeneExtensions 
  
   calculateHitsPerRound()   - 
Method in class util.bio.xamer. Xamer 
  
   calculateMaxCluster(MotifHit[], int)   - 
Static method in class meme. MotifScanner 
 Calculates the maximum number of hits that occur within a given window size.
   calculateMeanNumberOligoMatches(Oligo[])   - 
Static method in class trans.main. IntervalReportPrinter 
 Calculates the average number of oligo matches to the genome.
   calculateMedianOfChromosome(Quantile[], String)   - 
Method in class trans.cel. QuantileNormalization 
 Looks in the tpmapInfo for the appropriate chromosome, extracts the intensities from the Quantile[], calculates a median.
   calculateMedians(UCSCGeneLine[])   - 
Method in class trans.roc. ScoreParsedBars 
 Calculates the median intensity for the oligos that lie under a gene.
   calculateMedianStatFlagValues()   - 
Method in class trans.qc. CelFileQualityControl 
 Calculates the median value for each stat flag
   calculateMiddle(String[])   - 
Method in class edu.utah.seq.parsers. Tag2Point 
 Using interbase coordinates so length = end - start.
   calculateMiddleIntergenicCoordinates(int, int)   - 
Static method in class util.gen. Num 
 Using interbase coordinates so length = end - start.
   calculateMolecularWtDNA(int[])   - 
Static method in class util.bio.calc. NucleicAcid 
 Calculates molecular weight of a DNA sequence given an int[] of gatcn's, no 5' phosphate
   calculateMolecularWtRNA(int[])   - 
Static method in class util.bio.calc. NucleicAcid 
 Calculates molecular weight of a RNA sequence given an int[] of gaucn's, no 5' phosphate
   calculateNumberControlERs(float)   - 
Method in class edu.utah.seq.analysis. ScanSeqs 
 Given a score threshold, returns the number of EnrichedRegions that would be generated using the
 precomputed values.
   calculatePercentHitsPerRound(int[])   - 
Method in class util.bio.xamer. Xamer 
  
   calculatePoissonPValues(int, SmoothingWindow[], int, int, double)   - 
Method in class edu.utah.seq.analysis. ScanSeqs 
 Makes a call to R to use it to calculate Bonferroni corrected Poisson pvalues.
   calculateRandomLabelPermutationConfidenceScores()   - 
Method in class trans.main. ScanChip 
 Takes the histogram of random permutation scores and calculates an uncorrected p-value and FDR for each real score.
   calculateRandomPermutationConfidenceScores()   - 
Method in class trans.main. ScanChromosomes 
 Takes the histogram of random permutation scores and calculates a p-value and FDR for each real score.
   calculateRandomPermutationConfidenceScores()   - 
Method in class trans.main. ScanChromosomesCNV 
 Takes the histogram of random permutation scores and calculates a p-value and FDR for each real score.
   calculateSaltAdjustedOligoTm(String)   - 
Static method in class util.bio.calc. NucleicAcid 
 Calculate salt adjusted Oligo Tm
   calculateSignedRankStatistics()   - 
Method in class trans.main. WilcoxonSignedRankTest 
 Calculates signed rank statistic S = sum of the ranks (aka WPlus) for WilcoxonSamplePairs with a 
 positive difference and WMinus for those with a negative difference.
   calculateStats()   - 
Method in class trans.qc. CelFileStats 
 Reloads intensites and calculates mean, coef var, percentiles.
   calculateTestStatisticZw()   - 
Method in class trans.main. WilcoxonSignedRankTest 
 Calculates test statistic Z from Lowry .
   calculateTotalNumberOfOligosPerRound(String[][])   - 
Method in class util.bio.xamer. XamerCounter 
  
   calculateTotalWindowIntersectionStats()   - 
Method in class trans.roc. RocWindowScanner 
 Calculate total # windows that intersect pos regions, no counting, ie one window overlaps two pos regions is counted once.
   calculateWilcoxon(float[], float[])   - 
Method in class edu.utah.seq.methylation454. StatMethylationData 
  
   calculateX(double)   - 
Method in class util.gen. LinearRegression 
 Calculate X given Y.
   calculateY(double)   - 
Method in class util.gen. LinearRegression 
 Calculate Y given X.
   calculateZ()   - 
Method in class trans.main. WilcoxonRankSumTest 
 Only valid when # treatment samples and # control samples >9 each.
   canAlter(UserBean, String, int)   - 
Method in class bioroot. DBUtil 
 Checks whether the user can modify or delete an item.
   canModifyAll(UserBean)   - 
Method in class bioroot. ReagentBean 
  
   canModifyCommon(UserBean)   - 
Method in class bioroot. ReagentBean 
  
   canView(UserBean, String, int)   - 
Method in class bioroot. DBUtil 
 Checks whether the user can view an item without creating a bean, just an id
   canView(UserBean, String, String[])   - 
Method in class bioroot. DBUtil 
 Checks whether the user can view multiple items based on id.
   canView(UserBean, DBUtil)   - 
Method in class bioroot. ReagentBean 
  
   Cap3Parser   - Class in  util.bio.parsers      Cap3Parser(String[])   - 
Constructor for class util.bio.parsers. Cap3Parser 
  
   capitalizeFirstLetter(String)   - 
Static method in class util.gen. Misc 
 Capitalizes the first letter in a String.
   CelFileConverter   - Class in  trans.cel  Saves text cel files as serialized float[][]s for downstream use by TiMAT applications.   CelFileConverter(String[])   - 
Constructor for class trans.cel. CelFileConverter 
  
   CelFileQualityControl   - Class in  trans.qc  Provides automated qc checking for cel files, flags those of poor quality 
 based on a variety of statistics.   CelFileQualityControl()   - 
Constructor for class trans.qc. CelFileQualityControl 
  
   CelFileStats   - Class in  trans.qc  Container for cel file and it's associated statistics.   CelFileStats(File, String)   - 
Constructor for class trans.qc. CelFileStats 
 Loads virtualCel float[][] and calculates a rawMedian.
   CelLine   - Class in  trans.cel  Class for holding info about a text cel file line, enables sorting after rotation.   CelLine(int, int, String)   - 
Constructor for class trans.cel. CelLine 
  
   CellType   - Class in  bioroot      CellType()   - 
Constructor for class bioroot. CellType 
  
   CellTypeBean   - Class in  bioroot      CellTypeBean()   - 
Constructor for class bioroot. CellTypeBean 
  
   CellTypeBean(int, DBUtil)   - 
Constructor for class bioroot. CellTypeBean 
  
   CelMapper   - Class in  trans.cel  Saves float[]s of intensity values for each cel file ordered by the tpmapFeature file.   CelMapper(MapFeature[], boolean, boolean)   - 
Constructor for class trans.cel. CelMapper 
  
   CelMasker   - Class in  trans.graphics  Application to draw and mask a raw text cel file.   CelMasker(String[])   - 
Constructor for class trans.graphics. CelMasker 
  
   CelPHistogram   - Class in  trans.cel      CelPHistogram()   - 
Constructor for class trans.cel. CelPHistogram 
  
   CelProcessor   - Class in  trans.cel  Application to process '.cela' files, wrapper for   CelMapper  , 
   QuantileNormalization  .   CelProcessor(String[])   - 
Constructor for class trans.cel. CelProcessor 
  
   cgar   - package cgar     cgar.beans   - package cgar.beans     changeQuality(String, int, int)   - 
Static method in class selex. SeqFiles 
 Modifies a quality file, adding or subtracting scores.
   ChartFrame   - Class in  trans.qc  Frame for JFree Panels.   ChartFrame(JFreeChart[], String, int, int)   - 
Constructor for class trans.qc. ChartFrame 
  
   checkAlignParams(AlignParams[])   - 
Static method in class gata.plotter. MultiAlignPanel 
 Checks to see if the same params were used used in making the alignments, note score cut off is not checked
   checkEstimateNumSubSeqs()   - 
Method in class selex. SeqRead 
  
   checkFields()   - 
Method in class gata.aligner. AlignerInputPanel 
  
   checkFile(String)   - 
Static method in class gata.main. GATAUtil 
  
   checkFile(String)   - 
Static method in class meme. UtilMeme 
  
   checkFile(String)   - 
Static method in class util.gen. IO 
 Check to see that a file exists and is not a directory.
   checkFiles()   - 
Method in class util.bio.wrappers. Segment 
  
   checkForFlippedControls()   - 
Method in class trans.qc. CelFileQualityControl 
 Runs through groups of cel files, calculates the median no Synth, Dim, and Bright intensities,
 checks to see if they are inverted, for some reason they sometimes are!  Note these medians 
 are calculated on the raw, pre scaled chip intensities.
   checkIP(String)   - 
Method in class bioroot. DBUtil 
 Checks to see if a given IP has exceeded the number of allowable failed logins.
   checkIP(String)   - 
Method in class cgar. Util 
 Checks to see if a given IP has exceeded the number of allowable failed logins.
   checkMenu(String[])   - 
Method in class util.apps.gump. Gump 
  
   checkMenuParsing()   - 
Method in class util.apps.gump. Gump 
  
   checkNumberOfReplicas(File[][])   - 
Method in class trans.main. T2Parameter 
  
   checkParams(DBUtil)   - 
Method in class bioroot. LoginBean 
  
   checkParams()   - 
Method in class bioroot. UserRegistrationBean 
  
   checkParams(SQL)   - 
Method in class cgar.beans. LoginBean 
  
   checkPlasmidDBFile(DBUtil)   - 
Static method in class bioroot. Search 
 Looks for an upto date blast database file.
   checkScore(SmoothingWindow)   - 
Method in class edu.utah.seq.analysis. EnrichedRegionMaker 
  
   checkScoreAndNumOligos(Window)   - 
Method in class trans.main. IntervalMaker 
  
   checkSeqQuality(int[], int, int)   - 
Static method in class selex. SeqRead 
 Examines an int[] for a continuous stretch of scores
   checkSingleCelFileStats()   - 
Method in class trans.qc. CelFileQualityControl 
 Runs through groups of cel files, calling statSingleCelFile() on each
 and prints the results.
   checkSizes(float[][])   - 
Static method in class trans.main. MultiSGRPrinter 
 Checks to see if the number of values are the same given float[replica#][values].
   checkSizes(float[][])   - 
Static method in class trans.main. ScanChromosomes 
 Checks to see if the number of values are the same given float[replica#][values].
   checkSizes(float[][])   - 
Static method in class trans.main. ScanChromosomesCNV 
 Checks to see if the number of values are the same given float[replica#][values].
   checkUpdateParams(String)   - 
Method in class bioroot. UserRegistrationBean 
 Used when checking params for AccountManagement Update
   checkWidth(int, int, int)   - 
Method in class trans.main. PeakPicker 
  
   checkWindowScoresAndNumberOfOligos()   - 
Method in class trans.main. MultiWindowIntervalMaker 
 Checks the scores and number of oligos in each window.
   chrLetter   - 
Static variable in class util.bio.seq. Seq 
  
   chrNumber   - 
Static variable in class util.bio.seq. Seq 
  
   chromosome   - 
Variable in class trans.tpmap. MapFeature 
  
   ChromosomeIndex   - Class in  trans.cel  Use to keep track of intensities in a array, their chromosome name, and start stop indexes.   ChromosomeIndex(String, int, int)   - 
Constructor for class trans.cel. ChromosomeIndex 
  
   ChromSet   - Class in  trans.main      ChromSet()   - 
Constructor for class trans.main. ChromSet 
  
   cleanUpHash(int)   - 
Method in class trans.misc. InefficientPseudoMedian 
 Removes Pairs from the hash or reinstantiates if not needed.
   clearDups(ArrayList)   - 
Method in class gata.aligner. LocalAlignment 
  
   clearLines()   - 
Method in class gata.main. AnnoSpecParams 
 Wipes all the Line2D.Double ArrayLists, call before reDrawing
 the Glyphs when params have been changed.
   clearScaleLineArrays()   - 
Method in class gata.geneGlyphs. ScaleBar 
 Used in hiding scale ruler bar
   clipUnderScore(String)   - 
Static method in class util.gen. Misc 
 Removes the random digit_ proceeding a string, (ie 2432344322_fileName.txt to fileName.txt)
   clone()   - 
Method in class util.bio.parsers.gff. Gff3Feature 
  
   clone()   - 
Method in class util.gen. MD5 
  
   closeConnection()   - 
Method in class bioroot. DBUtil 
 Closes the Connection and subsequently the Statement and ResultSet objects
   closeConnection()   - 
Method in class util.gen. SQL 
 Closes the Connection and subsequently the Statement and ResultSet objects
   closeSetAmplicons()   - 
Method in class edu.utah.seq.methylation454. MethylationParser 
  
   closeSetCpGs()   - 
Method in class edu.utah.seq.methylation454. MethylationParser 
  
   closeSetReads()   - 
Method in class edu.utah.seq.methylation454. MethylationParser 
  
   cluster(int[][])   - 
Method in class trans.qc. CelFileQualityControl 
 Clusters cel files based on pm intensities by group.
   Cluster   - Class in  util.bio.cluster      Cluster(File, String)   - 
Constructor for class util.bio.cluster. Cluster 
 Initializing Constructor
   Cluster(Cluster, Cluster, double, StringBuffer)   - 
Constructor for class util.bio.cluster. Cluster 
 Secondary Constructor
   cluster()   - 
Method in class util.bio.cluster. HierarchicalClustering 
  
   clusterCelaFiles(File[], int[][])   - 
Method in class util.bio.cluster. HierarchicalClustering 
 Clusters cela files, extracts PM intensities, median normalizes.
   clusterCelFiles(int[][])   - 
Method in class trans.qc. CelFileQualityControl 
 Runs through groups of cel files, calling statSingleCelFile() on each
 and prints the results.
   ClusterDrawFrame   - Class in  util.bio.cluster  Helper class for   HierarchicalClustering  , the frame.   ClusterDrawFrame(HierarchicalClustering)   - 
Constructor for class util.bio.cluster. ClusterDrawFrame 
  
   ClusterDrawPanel   - Class in  util.bio.cluster  Helper class for   HierarchicalClustering  , the guts of drawing the clustering plot.   ClusterDrawPanel(HierarchicalClustering)   - 
Constructor for class util.bio.cluster. ClusterDrawPanel 
  
   clusterNormalizedFloatArrayFiles()   - 
Method in class util.bio.cluster. HierarchicalClustering 
 Clusters float[] files, does not median normalize
   clusterVirtualCelFiles(File[], int[][])   - 
Method in class util.bio.cluster. HierarchicalClustering 
 Performs actual clustering for CelFileQualityControl app.
   collapseDoubleArray(double[][])   - 
Static method in class util.gen. Num 
 Assumes equal lengths of the double[]s
   collapseExons()   - 
Method in class util.bio.parsers.gff. ParseWormBaseGffToUCSC 
  
   collapseFileArray(File[][])   - 
Static method in class util.gen. IO 
 Merges all files in File[][] to a File[].
   collapseFloatArray(float[][])   - 
Static method in class util.gen. Num 
 Assumes equal lengths of the float[]s
   collapseIntArray(int[][])   - 
Static method in class util.gen. Num 
 Any length OK of the int[]s
   collapseIntensityFeatureArray(IntensityFeature[][])   - 
Static method in class trans.cel. MakeChromosomeSets 
 Collapses an uneven array of IntensityFeature[][] to IntensityFeature[].
   collapsePartOfAnArray(float[][], int, int)   - 
Static method in class util.gen. Num 
 Assumes equal lengths of the float[]s
   CollapseTPMap   - Class in  trans.tpmap  Collapses a text version bpmap into a unique set of bpmap lines base on the oligo sequence.   CollapseTPMap()   - 
Constructor for class trans.tpmap. CollapseTPMap 
  
   colorizeGenericGlyphs(int)   - 
Method in class gata.menu. GATAFrame 
 helper method, 0 = linear, 1 = log10, 2 = lnx, 3= reset to solid 4= log2
   colorizeTrackedGenericGlyphs(int)   - 
Method in class gata.geneGlyphs. Annotation 
 This method converts scores in generic glyphs to changes in alpha, the opacity 
 number for the glyph color.
   COLORS   - 
Static variable in class gata.main. GATAUtil 
 Color array for tracks, yup I'm lazy
   combinePointData(ArrayList<PointData>, boolean)   - 
Static method in class edu.utah.seq.data. PointData 
 Combines without summing the scores, thus duplicate positions are likely.
   CombineXYSeries   - Class in  trans.graphics      CombineXYSeries()   - 
Constructor for class trans.graphics. CombineXYSeries 
  
   ComparatorEnrichedRegionPosition   - Class in  edu.utah.seq.data      ComparatorEnrichedRegionPosition()   - 
Constructor for class edu.utah.seq.data. ComparatorEnrichedRegionPosition 
  
   ComparatorEnrichedRegionScore   - Class in  edu.utah.seq.data      ComparatorEnrichedRegionScore(int)   - 
Constructor for class edu.utah.seq.data. ComparatorEnrichedRegionScore 
  
   ComparatorInfoPoint   - Class in  edu.utah.seq.data  Class to sort Info by chromosome.   ComparatorInfoPoint()   - 
Constructor for class edu.utah.seq.data. ComparatorInfoPoint 
  
   ComparatorMultiSetQuantile   - Class in  trans.cel  For sorting MultiSetQuantile objects by value or chromosome number and position   ComparatorMultiSetQuantile(boolean)   - 
Constructor for class trans.cel. ComparatorMultiSetQuantile 
 Set boolean sortByValue = true to sort by value, false to sort by chromosome number and position.
   ComparatorPointPosition   - Class in  edu.utah.seq.data  Class to help sort an array of Window[] by chromosome, start position, length shortest first.   ComparatorPointPosition()   - 
Constructor for class edu.utah.seq.data. ComparatorPointPosition 
  
   ComparatorQuantile   - Class in  trans.cel  For sorting ComparatorQuantile objects by value or chromosome number and position   ComparatorQuantile(boolean)   - 
Constructor for class trans.cel. ComparatorQuantile 
 Set boolean sortByValue = true to sort by value, false to sort by chromosome number and position.
   ComparatorSmoothingWindowPosition   - Class in  edu.utah.seq.data      ComparatorSmoothingWindowPosition()   - 
Constructor for class edu.utah.seq.data. ComparatorSmoothingWindowPosition 
  
   ComparatorSmoothingWindowScore   - Class in  edu.utah.seq.data      ComparatorSmoothingWindowScore(int)   - 
Constructor for class edu.utah.seq.data. ComparatorSmoothingWindowScore 
  
   ComparatorSmoothingWindowTwoScore   - Class in  edu.utah.seq.data  Sorts by scores[0], then scores[1]   ComparatorSmoothingWindowTwoScore()   - 
Constructor for class edu.utah.seq.data. ComparatorSmoothingWindowTwoScore 
  
   compare(Object, Object)   - 
Method in class edu.utah.seq.data. ComparatorEnrichedRegionPosition 
 Sorts by chromosome and position
   compare(Object, Object)   - 
Method in class edu.utah.seq.data. ComparatorEnrichedRegionScore 
 Sorts by score, largest to smallest
   compare(Object, Object)   - 
Method in class edu.utah.seq.data. ComparatorInfoPoint 
 Sorts by name
   compare(Object, Object)   - 
Method in class edu.utah.seq.data. ComparatorPointPosition 
 Sorts by position
   compare(Object, Object)   - 
Method in class edu.utah.seq.data. ComparatorSmoothingWindowPosition 
 Sorts by start position, smallest to largest
   compare(Object, Object)   - 
Method in class edu.utah.seq.data. ComparatorSmoothingWindowScore 
 Sorts by score, smallest to largest
   compare(Object, Object)   - 
Method in class edu.utah.seq.data. ComparatorSmoothingWindowTwoScore 
  
   compare(GeneGroup)   - 
Method in class trans.anno. BindingRegion 
 Compares a binding region to a gene grp looking for where it is in relation to the binding 
 region, 5', 3', overlap, contained.
   compare(Object, Object)   - 
Method in class trans.anno. BindingRegionComparator 
 Sorts by rank
   compare(Object, Object)   - 
Method in class trans.anno. RegionComparator 
 Sorts by chromosome-> start oligo position -> length, shortest first
   compare(Object, Object)   - 
Method in class trans.cel. ComparatorMultiSetQuantile 
 Sorts by value (default) or chipNumber and position based on sortByValue boolean, all smallest to biggest.
   compare(Object, Object)   - 
Method in class trans.cel. ComparatorQuantile 
 Sorts by value (default) or position based on sortByValue boolean, all smallest to biggest.
   compare(Object, Object)   - 
Method in class trans.main. IntervalComparator 
 Sorts by chromosome-> start oligo position -> length, shortest first
   compare(Object, Object)   - 
Method in class trans.main. WindowComparator 
 Sorts by chromosome-> start oligo position -> length, shortest first
   compare(Object, Object)   - 
Method in class trans.misc. SgrComparator 
 Sorts by chromosome then position
   compare(Object, Object)   - 
Method in class trans.roc. GrComparator 
 Sorts by start position
   compare(Object, Object)   - 
Method in class trans.roc. PositiveComparator 
 Sorts by chromosome-> start oligo position -> length, shortest first
   compare(Object, Object)   - 
Method in class util.bio.parsers. UCSCGeneLineComparator 
 Sorts by chromosome-> start position -> length, shortest first
   compare(Object, Object)   - 
Method in class util.bio.parsers. UCSCGeneLineComparatorScore 
  
   compare(Object, Object)   - 
Method in class util.gen. RankSampleIndexComparator 
  
   compare(Object, Object)   - 
Method in class util.gen. RankSampleValueComparator 
  
   compareBindingRegionsVsGeneGrps(GeneGroup[], BindingRegion[])   - 
Static method in class trans.anno. AnnotateRegions 
 Does a complete scan, could be optimized.
   compareBindingRegionsVsGeneGrps(GeneGroup[], BindingRegion[])   - 
Static method in class trans.anno. AnnotateRegionsWithGeneList 
 Does a complete scan, could be optimized.
   compareTo(Object)   - 
Method in class bioroot.antibody. AntibodyBean 
  
   compareTo(Object)   - 
Method in class bioroot. BlastMatcher 
  
   compareTo(Object)   - 
Method in class bioroot. Feature 
 For sorting by order.
   compareTo(Object)   - 
Method in class bioroot.plasmid. PlasmidBean 
  
   compareTo(Object)   - 
Method in class bioroot. ReagentBean 
  
   compareTo(Object)   - 
Method in class bioroot. SearchHit 
 Sorts by score
   compareTo(Object)   - 
Method in class bioroot.strain. StrainBean 
  
   compareTo(Object)   - 
Method in class bioroot. TableHeading 
 For sorting
   compareTo(Object)   - 
Method in class edu.utah.seq.methylation454. DataLine 
  
   compareTo(Object)   - 
Method in class expr. ExpressedGene 
 Sorts by Coordinate
   compareTo(Object)   - 
Method in class gata.aligner. Alignment 
  
   compareTo(Object)   - 
Method in class gata.plotter. StartStop 
  
   compareTo(Object)   - 
Method in class meme. MotifHit 
  
   compareTo(Object)   - 
Method in class meme. MotifSearchResult 
  
   compareTo(Object)   - 
Method in class trans.anno. BindingRegion 
  
   compareTo(Object)   - 
Method in class trans.bkgrnd. LoadedOligo 
  
   compareTo(Object)   - 
Method in class trans.cel. CelLine 
 Sort first by y coordinate, then x.
   compareTo(Object)   - 
Method in class trans.main. Interval 
  
   compareTo(Object)   - 
Method in class trans.main. PosRegion 
  
   compareTo(Object)   - 
Method in class trans.main. ScoredGene 
  
   compareTo(Object)   - 
Method in class trans.main. WilcoxonSample 
  
   compareTo(Object)   - 
Method in class trans.main. WilcoxonSamplePair 
  
   compareTo(Object)   - 
Method in class trans.main. Window 
  
   compareTo(Object)   - 
Method in class trans.misc. GCRatio 
  
   compareTo(Object)   - 
Method in class trans.roc. RocWindow 
  
   compareTo(Object)   - 
Method in class trans.tpmap. IntensityFeature 
 Sorts by  position.
   compareTo(Object)   - 
Method in class trans.tpmap. MapFeature 
 Sorts by chromosome and then position.
   compareTo(Object)   - 
Method in class trans.tpmap. TPMapLine 
  
   compareTo(Object)   - 
Method in class util.bio.annotation. Coordinate 
 Sorts by chromsome, start position, length (smallest to largest).
   compareTo(Object)   - 
Method in class util.bio.annotation. ExonIntron 
  
   compareTo(Object)   - 
Method in class util.bio.annotation. GeneGroup 
  
   compareTo(Object)   - 
Method in class util.bio.annotation. GenericFeature 
  
   compareTo(Object)   - 
Method in class util.bio.annotation. StartStop 
  
   compareTo(Object)   - 
Method in class util.bio.digest. Enzyme 
  
   compareTo(Object)   - 
Method in class util.bio.parsers. Fasta 
  
   compareTo(Object)   - 
Method in class util.bio.parsers.gff. Gff3Feature 
 Sorts by the sortBy field so set it before sorting.
   compareTo(Object)   - 
Method in class util.bio.seq. IndexedSequence 
 Assumes starts are unique.
   compareTo(Object)   - 
Method in class util.gen. FileSize 
  
   complementDNA(String)   - 
Static method in class util.bio.seq. Seq 
 Takes a DNA seq and complement it, ambiguous symbols OK.
   completeAnnotationMenu(boolean, boolean, AnnoSpecParams, AnnoSpecParams)   - 
Method in class gata.menu. GATAFrame 
 must be called after the initial creation when the user has possibly loaded generic annotation
   componentHidden(ComponentEvent)   - 
Method in class util.apps.gump. GumpApplicationPanel 
  
   componentMoved(ComponentEvent)   - 
Method in class util.apps.gump. GumpApplicationPanel 
  
   componentResized(ComponentEvent)   - 
Method in class util.apps.gump. GumpApplicationPanel 
  
   componentShown(ComponentEvent)   - 
Method in class util.apps.gump. GumpApplicationPanel 
  
   CompressFasta   - Class in  edu.utah.seq.compression  Application that compresses or decompresses a multi fasta file.   CompressFasta(File[])   - 
Constructor for class edu.utah.seq.compression. CompressFasta 
  
   compressIt(File, File, String)   - 
Method in class edu.utah.seq.compression. CompressFasta 
  
   concatinate()   - 
Method in class trans.main. RandomizeLabels 
 Concat name numbers to a String.
   concatinate(float[][])   - 
Static method in class util.gen. Num 
 Concatinates the float[]s, variable sizes OK.
   concatinate(float[], float[])   - 
Static method in class util.gen. Num 
 Joins two float[]s using System.arraycopy().
   concatinateFileFullPathNames(File[], String)   - 
Static method in class util.gen. IO 
 Concatinates the full path file names for the given File array.
   connect2Db(String, String, String)   - 
Method in class util.gen. SQL 
 Method to connect to the database and create a statement object
   connectAndTest()   - 
Method in class util.amazon. S3Manager 
 Instantiates connection objects and attempt to make a bucket if it doesn't exist to hold objects.
   connected   - 
Variable in class util.amazon. S3Manager 
  
   connectMySQL(String)   - 
Method in class bioroot. DBUtil 
 Method to connect to the MySQL lab_collection database
 and create a statement object
   ConservedSeqs   - Class in  gata.plotter      ConservedSeqs(GATAParams)   - 
Constructor for class gata.plotter. ConservedSeqs 
  
   Console   - Class in  gata.plotter      Console(int, int, int, int, String)   - 
Constructor for class gata.plotter. Console 
  
   Contact   - Class in  bioroot      Contact()   - 
Constructor for class bioroot. Contact 
  
   ContactBean   - Class in  bioroot      ContactBean()   - 
Constructor for class bioroot. ContactBean 
  
   contactEmail   - 
Static variable in class bioroot. Util 
 Who do you call?
   contactEmail   - 
Static variable in class cgar. Util 
  
   contained(int)   - 
Method in class util.bio.seq. IndexedSequence 
  
   containedWithin(BindingRegion, BindingRegion)   - 
Static method in class trans.anno. IntersectRegions 
 Returns true if two entirely contains one.
   contains(double)   - 
Method in class trans.roc. Bin 
  
   contains(int)   - 
Method in class trans.roc. Positive 
  
   contains(double)   - 
Method in class util.gen. Bin 
 Returns true if the value is >= bin.start and    containsMatchedRankIntersect(GenomicRegion, int)   - 
Static method in class trans.graphics. RankedSetAnalysis 
 Checks to see if among the hits to a particular GenomicRegion they have an equal or smaller rank.
   containsPoint(int)   - 
Method in class trans.roc. ControlGene 
 Looks to see if point is contained in any of the exons, ends are inclusive.
   controlChromosomeName   - 
Static variable in class trans.tpmap. MummerMapper 
 String given to naming the control sequences.
   ControlGene   - Class in  trans.roc      ControlGene(String)   - 
Constructor for class trans.roc. ControlGene 
  
   ControlStats   - Class in  trans.cel      ControlStats()   - 
Constructor for class trans.cel. ControlStats 
  
   convert()   - 
Method in class trans.cel. CelFileConverter 
  
   convert(File)   - 
Static method in class trans.tpmap. ConvertTPMapsToFeatures 
 Converts a text bpmap to a MapFeature[]
   convert2Boolean(byte[])   - 
Method in class util.bio.seq. BinaryBooleanReaderWriter 
  
   convert2Bytes(boolean[])   - 
Method in class util.bio.seq. BinaryBooleanReaderWriter 
 Assumes the boolean[] is evenly divisable by 8.
   convert2String(boolean[], int, int)   - 
Static method in class util.bio.seq. BinaryBooleanReaderWriter 
 Converts a boolean[] to a 0=false,1=true String.
   ConvertAgilentData   - Class in  trans.misc  Reads in an Agilent tiling data file and outputs a tpmap and four cela files.   ConvertAgilentData(String[])   - 
Constructor for class trans.misc. ConvertAgilentData 
  
   ConvertBars2TPMapCel   - Class in  trans.misc  Reads in multiple directories of bar files and makes xxx.cela and xxx.tpmap files.   ConvertBars2TPMapCel(String[])   - 
Constructor for class trans.misc. ConvertBars2TPMapCel 
  
   convertBitScoreToRaw(double, double, double)   - 
Static method in class gata.main. GATAUtil 
  
   convertBitScoreToRaw(double, double, double)   - 
Static method in class util.bio.calc. Alignment 
 Converts a nat bit score to raw S.
   convertCelFiles()   - 
Method in class trans.main. T2 
  
   ConvertFasta2GCBoolean   - Class in  util.bio.seq  Converts fasta file(s) into serialized boolean[]s where every base g or c is true all others false.   ConvertFasta2GCBoolean(String[])   - 
Constructor for class util.bio.seq. ConvertFasta2GCBoolean 
  
   ConvertFasta2RealSeqBoolean   - Class in  util.bio.seq  Converts fasta file(s) into compressed binary boolean[]s where GATC is true all (NXgatcnx) others false.   ConvertFasta2RealSeqBoolean(String[])   - 
Constructor for class util.bio.seq. ConvertFasta2RealSeqBoolean 
  
   ConvertGeoData   - Class in  trans.misc  Reads and parses in a GEO data file into a tpmap and xxx.cela files for TiMAT2 processing.   ConvertGeoData(String[])   - 
Constructor for class trans.misc. ConvertGeoData 
  
   ConvertNimblegenNDF2TPMap   - Class in  trans.misc  Converts a Nimblegen NDF txt file to a tpmap.!   ConvertNimblegenNDF2TPMap(String[])   - 
Constructor for class trans.misc. ConvertNimblegenNDF2TPMap 
  
   ConvertNimblegenPAIR2Cela   - Class in  trans.misc  Converts Nimblegen PAIR txt files to a cela files.!   ConvertNimblegenPAIR2Cela(String[])   - 
Constructor for class trans.misc. ConvertNimblegenPAIR2Cela 
  
   convertNumOrientationToStrand(int)   - 
Static method in class util.bio.annotation. GeneGroup 
  
   convertOrganismIdToName(int)   - 
Method in class bioroot. DBUtil 
 Converts and Organism id to and Organism name.
   convertOrganismNameToId(String)   - 
Method in class bioroot. DBUtil 
 Converts and Organism name to and OrganismId.
   convertPixelXCoorToBP(double)   - 
Method in class trans.graphics. IntervalDrawPanel 
 Uses pixelsPerBase and X from first Graph to convert a given X Cooridinate to base pairs.
   convertPlusToNumOrientation(String)   - 
Static method in class gata.main. GATAUtil 
  
   convertPlusToNumOrientation(String)   - 
Static method in class util.bio.annotation. GeneGroup 
  
   convertRatiosToPValues()   - 
Method in class trans.main. ScanChip 
  
   convertRatiosToPValues()   - 
Method in class trans.main. ScanChromosomes 
  
   convertRatiosToPValues()   - 
Method in class trans.main. ScanChromosomesCNV 
  
   convertRawScoreToBit(double, double, double)   - 
Static method in class gata.main. GATAUtil 
 Converts a raw S score to a bit score given lambda (in nats) and K
   convertRawScoreToBit(double)   - 
Method in class gata.plotter. ToolsFrame 
 Converts a raw S score to a bit score given lambda (in nats) and K
   convertRawScoreToBit(double, double, double)   - 
Static method in class util.bio.calc. Alignment 
 Converts a raw S score to a bit score given lambda (in nats) and K
   convertScores(String)   - 
Static method in class edu.utah.seq.parsers. ElandExportLine 
 Converts the ascii quality scores to numeric scores.
   convertTempFiles()   - 
Method in class edu.utah.seq.parsers. Tag2Point 
  
   convertToBED(Interval[])   - 
Method in class trans.main. IntervalGraphPrinter 
 Converts an array of Interval to a .bed String format.
   convertToBlocks(StartStop[])   - 
Static method in class gata.plotter. ConservedSeqs 
  
   convertToDouble(int[])   - 
Static method in class util.gen. Num 
 Convert to double[]
   convertToFloat(double[])   - 
Static method in class util.gen. Num 
 Converts a double[] to float[]
   convertToFloat(int[])   - 
Static method in class util.gen. Num 
 Convert to float[]
   convertToIndexPositions(ExonIntron[])   - 
Method in class trans.main. ScanGenes 
 Converts an array of ExonIntron to an int[exon number][start index in bp positions array, stop index]
   convertToInt(double[])   - 
Static method in class util.gen. Num 
 Convert to int[]
   convertToInt(float[])   - 
Static method in class util.gen. Num 
 Convert to int[]
   convertToMedianZScores(double[])   - 
Static method in class util.gen. Num 
 Converts an array of values to z scores using median as appose to mean.
   convertToMicroMolarDNA(String, double)   - 
Static method in class util.bio.calc. NucleicAcid 
 coverts ng/ul to uM given a DNA sequence
   convertToMicroMolarRNA(String, double)   - 
Static method in class util.bio.calc. NucleicAcid 
 coverts ng/ul to uM given an RNA sequence
   convertToNeg10Log10(double[])   - 
Static method in class util.gen. Num 
 Converts an array of double using -10 * (Math.log(double)/log10).
   convertToNgPerUlDNA(String, double)   - 
Static method in class util.bio.calc. NucleicAcid 
 coverts uM to ng/ul given a DNA sequence
   convertToNgPerUlRNA(String, double)   - 
Static method in class util.bio.calc. NucleicAcid 
 coverts uM to ng/ul given an RNA sequence
   convertToSGR(Interval[])   - 
Method in class trans.main. IntervalGraphPrinter 
 Converts an array of Interval to a .sgr String format.
   convertToZScores(double[], double, double)   - 
Static method in class util.gen. Num 
 Converts an array of values to z scores given their mean and standard deviation.
   convertToZScores(double[])   - 
Static method in class util.gen. Num 
 Converts an array of values to z scores.
   ConvertTPMapsToFeatures   - Class in  trans.tpmap  Converts TPMaps to MapFeature[] and saves them to disk.   ConvertTPMapsToFeatures(String[])   - 
Constructor for class trans.tpmap. ConvertTPMapsToFeatures 
  
   convertTPMapToFeatures(File)   - 
Method in class trans.tpmap. TPMapProcessor 
 Converts a text tpmap to a MapFeature[] and tracks duplicates
   ConvertTVSToCelps   - Class in  trans.cel  Converts a tsv file to xxx.celp files (float[]s) linked to the tpmap.   ConvertTVSToCelps(String[])   - 
Constructor for class trans.cel. ConvertTVSToCelps 
  
   Coordinate   - Class in  util.bio.annotation  Base class for holding info about a genomic coordinate.   Coordinate(String, int, int)   - 
Constructor for class util.bio.annotation. Coordinate 
  
   CoordinateExtractor1lq   - Class in  trans.qc  Extracts control an pm lines from a text 1lq file.   CoordinateExtractor1lq(String[])   - 
Constructor for class trans.qc. CoordinateExtractor1lq 
  
   copy(String)   - 
Method in class edu.utah.seq.data. FilterPointData 
  
   copy(File, File)   - 
Static method in class util.gen. IO 
 Fast & simple file copy.
   copyDirectoryRecursive(File, File, String)   - 
Static method in class util.gen. IO 
 Copies a given directory and it's contents to the destination directory.
   corrCoeff(float[], float[])   - 
Method in class util.gen. SpearmanCorrelation 
 This is the final step in calculating a Spearman Correlation coeff.
   correlate()   - 
Method in class edu.utah.seq.analysis. QCSeqs 
  
   correlate(HashMap<String, QCSeqs.ChromWindowScores>, HashMap<String, QCSeqs.ChromWindowScores>)   - 
Method in class edu.utah.seq.analysis. QCSeqs 
  
   correlate()   - 
Method in class expr. CorrelationMaps 
 Creates CorrelationWindows and performs random trials.
   Correlate   - Class in  util.apps  Calculates a Pearson correlation coefficient between all pairs of serializae float[].   Correlate(String[])   - 
Constructor for class util.apps. Correlate 
  
   Correlate(File[], File)   - 
Constructor for class util.apps. Correlate 
  
   correlate()   - 
Method in class util.apps. Correlate 
  
   CorrelateGrFiles   - Class in  trans.misc      CorrelateGrFiles()   - 
Constructor for class trans.misc. CorrelateGrFiles 
  
   correlationCoefficient(int[], int[])   - 
Static method in class util.gen. Num 
 Calculates Pearson correlation coefficient, r, from two int[]s.
   correlationCoefficient(float[], float[])   - 
Static method in class util.gen. PearsonCorrelation 
 Calculates Pearson correlation coefficient, r, from two float[]s.
   correlationCoefficient(double[], double[])   - 
Static method in class util.gen. PearsonCorrelation 
 Calculates Pearson correlation coefficient, r, from two double[]s
   CorrelationMaps   - Class in  expr      CorrelationMaps(String[])   - 
Constructor for class expr. CorrelationMaps 
  
   CorrelationWindow   - Class in  expr      CorrelationWindow(float, String, int, int, String[])   - 
Constructor for class expr. CorrelationWindow 
  
   count(double)   - 
Method in class util.gen. Bin 
 If value is contains() returns true and increments the bin hit counter, otherwise returns false.
   count(double, double)   - 
Method in class util.gen. Bin 
 If value is contains() returns true and increments the bin hit counter and total otherwise returns false.
   count(double)   - 
Method in class util.gen. Histogram 
 Increments the proper bin.
   count(double, double)   - 
Method in class util.gen. Histogram 
 Increments the proper bin.
   countAll(float[])   - 
Method in class util.gen. Histogram 
 Counts all the values incrementing the proper bin.
   countAll(double[])   - 
Method in class util.gen. Histogram 
 Counts all the values incrementing the proper bin.
   countAll(double[], double[])   - 
Method in class util.gen. Histogram 
 Counts all the values incrementing the proper bin and scores.
   countBase(char)   - 
Method in class util.bio.parsers. BaseCount 
 Base must be lower case, if not gatc then n is incremented.
   countBases(String)   - 
Static method in class util.bio.seq. Seq 
 Given a sequence returns the number of G's,A's,T's,C's,N's as an int[5].
   countDataLines(File)   - 
Method in class trans.misc. ConvertGeoData 
  
   countEnrichedRegions(boolean, float)   - 
Method in class edu.utah.seq.analysis. EnrichedRegionMaker 
 Returns the number of ERs that would have been made.
   countGenes(BindingRegion[])   - 
Static method in class trans.anno. AnnotateRegions 
 Returns the number of genes where the binding region is on the 5' end and
 the number of genes where the binding region is on the 3' end of the respective gene,
 the number of genes that overlap a binding region on their 5' end and 3' end, lastly 
 the number of regions entirely contained by a gene,
 the number of binding regions with neighbors,
 the number of regions with no neighbors as defined by the neighborhood,
 the number of regions in non coding DNA,
 the number of regions in coding DNA,
 the number of regions that overlap coding and nonCoding DNA
   countGenes(BindingRegion[])   - 
Static method in class trans.anno. AnnotateRegionsWithGeneList 
 Returns:
 the number of genes with one or more binding regions within the neighborhood.
   countLengths(Region[])   - 
Method in class trans.anno. RandomRegions 
  
   countNonBlankLines(File)   - 
Static method in class util.gen. IO 
 Counts the number of lines in a file skipping blanks.
   countNumberBindingRegionsWithNeighbors(BindingRegion[])   - 
Static method in class trans.anno. AnnotateRegions 
  
   countNumberBindingRegionsWithNeighbors(BindingRegion[])   - 
Static method in class trans.anno. AnnotateRegionsWithGeneList 
  
   countNumberIntensities(int[][][])   - 
Static method in class trans.qc. CoordinateExtractor1lq 
 Calculate number of noSynth, dim, bright controls, and pm probes.
   countNumberIntensities(int[][][])   - 
Static method in class trans.qc. Parser1lq 
 Calculate number of noSynth, dim, bright controls, and pm probes.
   countNumberNeighbors(BindingRegion[])   - 
Static method in class trans.anno. AnnotateRegions 
  
   countNumberNeighbors(BindingRegion[])   - 
Static method in class trans.anno. AnnotateRegionsWithGeneList 
  
   countNumberOfLines(File)   - 
Static method in class util.gen. IO 
 Counts the number of lines in a file
   countNumberOfOligoPositions(int[][])   - 
Static method in class trans.misc. Util 
 Given an array of int[][start, stop] returns the total number of integers, stop included.
   countNumberThatExist(File[])   - 
Static method in class util.gen. IO 
 Counts the number of File in the array that actually exist.
   countNumberTrue(boolean[])   - 
Static method in class util.gen. Num 
  
   countObjects(Object[][])   - 
Static method in class util.gen. Num 
 Returns the number of objects in the arrays.
   countOutliers(float[], double, boolean)   - 
Static method in class util.gen. Num 
 Counts the number of values that are > or    countTrues(boolean[], int, int)   - 
Static method in class util.gen. Misc 
 Does not check if indexes are out of bounds!
   countValues(int[][])   - 
Static method in class util.gen. Num 
 Returns the number of elements/ values in the array.
   CpG   - Class in  edu.utah.seq.methylation454      CpG(String)   - 
Constructor for class edu.utah.seq.methylation454. CpG 
  
   createCelFileStatsArray()   - 
Method in class trans.qc. CelFileQualityControl 
 Creates a cel file stats array, then saves and nulls the float arrays.
   createFeatureConcat(Feature[])   - 
Static method in class bioroot. Feature 
 Creates a String concatinate from a Feature[] for storage in the database.
   createHashMap(String[])   - 
Static method in class util.gen. Misc 
 Creates a HashMap from a String[] where the array is ordered such as key,value,key,value...
   createLinkedHashMap(String[], int)   - 
Static method in class util.gen. Misc 
 Creates a LinkedHashMap from a String[] where the array is ordered such as key,value,key,value...
   createRandomWords(String[], int, int)   - 
Static method in class util.gen. Passwords 
 Creates pseudorandom Strings derived from an alphabet of String[] using the
 java.util.Random class.
   createRandowWord(int)   - 
Static method in class util.gen. Passwords 
 Returns a random word using nonambiguous alphabet.
   createSamplePairs(float[], float[], boolean)   - 
Method in class trans.main. WilcoxonSignedRankTest 
 Creates WilcoxonSamplePair objects from two float[]s.
   createShapes(Alignment[], double, double, double, double, double, double, double)   - 
Static method in class gata.plotter. AlignPanel 
  
   createStringAL(String[])   - 
Static method in class meme. UtilMeme 
  
   createUserBean(DBUtil, String, String)   - 
Method in class bioroot. LoginBean 
  
   createVirtualCel(File)   - 
Static method in class trans.misc. Util 
 Loads a text version cel file into a zeroed virtual chip.
   createVirtualCel(String[])   - 
Static method in class trans.misc. Util 
 Loads a text version cel file into a zeroed virtual chip.
   credentialsFile   - 
Variable in class util.amazon. S3Manager 
  
   CronArchive   - Class in  bioroot  Emails lab group's collections to superusers.   CronArchive()   - 
Constructor for class bioroot. CronArchive 
  
   crypt(String, String)   - 
Static method in class util.gen. MD5Crypt 
  
   CubicSpline   - Class in  util.gen  For calculating cubic splines of incremental data, interpolation, from http://www.frank-buss.de/CubicSpline.java   CubicSpline(double[])   - 
Constructor for class util.gen. CubicSpline 
 The constructor calculates the second derivatives of the interpolating function
 at the tabulated points xi, with xi = (i, y[i]).
 
 


 
   
  
 
 
 
   
 
   
          Overview     
        Package   
        Class   
        Use   
          Tree     
          Deprecated     
       Index    
          Help     
   
 
 
  
 
 
 

 
  
   PREV LETTER   
   NEXT LETTER    
  
    FRAMES     
   NO FRAMES     
  
    All Classes  ');
  }
  //-->
 
 
    All Classes  
 


  
 
 
  
 

 A   B   C   D   E   F   G   H   I   J   L   M   N   O   P   Q   R   S   T   U   V   W   X   Z
